# “It’s all About the Colors:” How do Mexico City Youth Perceive Cigarette Pack Design

**DOI:** 10.3389/ijph.2021.585434

**Published:** 2021-03-10

**Authors:** Graziele Grilo, Lisa P. Lagasse, Joanna E. Cohen, Meghan B. Moran, Luz Myriam Reynales-Shigematsu, Katherine C. Smith

**Affiliations:** ^1^ Department of Health, Behavior and Society, Institute for Global Tobacco Control, Johns Hopkins Bloomberg School of Public Health, Baltimore, MD, United States; ^2^ Department of Health, Behavior and Society, Johns Hopkins Bloomberg School of Public Health, Baltimore, MD, United States; ^3^ Departamento de Prevención y Control de Tabaquismo, Centro de Investigación en Salud Poblacional, Instituto Nacional de Salud Pública, Cuernavaca, Mexico

**Keywords:** global tobacco control, product packaging, marketing, youth, qualitative research, flavor capsule

## Abstract

**Objectives:** Cigarette packs are relevant to branding strategies, designed to appeal to specific groups. There is little research on how pack features increase product appeal among key constituents such as youth in low- and middle-income countries.

**Methods:** We conducted 10 focus group discussions (FGDs) with adolescents and 5 FGDs with young adult smokers in Mexico City, separated by age, gender, smoking, and socioeconomic status. Participants separated 23 cigarette packs into “appealing” and “unappealing” groups, and were asked to explain their decisions, describing the features that supported their views. FGDs were video-recorded, transcribed in Spanish, translated into English, and subjected to thematic analysis.

**Results:** Pack groupings did not differ greatly across FGDs; bold, contrasting colors and elements communicating flavor and promotion increased cigarette pack appeal and desire to try. Participants perceived packs with these features to be used by and designed for youth, like themselves.

**Conclusion:** Our findings reinforce the importance of packaging design in attracting new consumers and maintaining current ones. Mexico should consider stronger tobacco advertising policies that include packaging color and depiction of flavor to reduce product appeal.

## Introduction

Restricting tobacco advertising, promotion, and sponsorship (TAPS) is an effective tobacco control measure recommended by the World Health Organization’s Framework Convention on Tobacco Control [1]. As countries have adopted restrictions to traditional advertising media, such as television and billboards, cigarette packs have become a valuable communication platform [2]. Not only are packs on display at the point-of-sale (POS) encouraging purchasing and experimentation [3], but they are also carried around with users, disseminating brand imagery [2]. It is well established that the tobacco industry designs cigarette packs to target specific consumer groups, such as women and young adults [2]. Packs have also been designed to convey less harm and strength through color, for example, light colored packs such as white are usually perceived as milder especially when compared with red packs [2]. The addition of flavors to tobacco is another way to advertise a less harmful product [4]. Through packaging design, flavored cigarettes can be identified using specific names, images, colors, and flavor capsules (a capsule in the filter that releases flavor when pressed). Packaging has also been used to foster associations between the product and feelings of freedom, independence, and peer acceptance [4].

By prohibiting the use of logos, colors, brand imagery and promotion, plain packaging is a recognized tobacco control measure to prevent the use of packs as a marketing tool and to increase the effectiveness of health warning labels [5]. Overall, prior studies found that branded packs are deemed more appealing than plain ones, with color playing an important role in increasing appeal and/or communicating reduced harm or product strength [6–11]. Most existing work has been quantitative, leaving unexplored how specific design features of the packs might affect curiosity, appeal, and intentions to try the product among different groups. Recognizing these specific design features is important in countries like Mexico, which currently has a partial TAPS ban, allows packs to be displayed at the POS, and has a graphic health warning label that only covers 30% of the pack front [12], leaving enough space for branding on the pack.

Mexico has observed a decrease in smoking prevalence since 2004 [13]. However, 27.5% of never-smoker adolescents (13–15 years) are likely to initiate cigarette smoking in the upcoming year [14]; in addition, 4.9% of Mexican adolescents (12–17 years) and 28.5% of the young adult population (18–24 years) currently smoke tobacco [15]. Understanding why specific design features are more appealing may elucidate the role of cigarette packaging in increasing youth smoking initiation, escalation, and brand commitment. To the best of our knowledge, this is the first study to use qualitative methods to examine how specific design features of available cigarette packs on the market contribute to product appeal among youth in a middle income country.

## Methods

### Study Design

We contracted with Berumen Y Asociados, a Mexican research company, to conduct small focus group discussions (FGDs) with adolescents and young adults in November 2018 in Mexico City, separated by gender (male, female), smoking status (smokers, non-smokers), and socioeconomic status (SES) (low, mid/high). Considering that tobacco use usually starts during adolescence [16], we included both smoking and non-smoking adolescents, but only recruited young adult smokers; non-smoking young adults were seen as less potentially susceptible to tobacco marketing.

After a pilot study conducted in Baltimore, United States, we determined that each FGD would ideally be comprised of four to six participants, allowing groups to be small enough so that all members would actively interact with one another and engage in the pack sorting activity, yet large enough that the groups would reflect a diversity of opinions on the topic. We anticipated that between 48 and 72 youth would take part in the study overall and that saturation would likely be achieved after 12 FGDs. A total of 56 participants attended one of 15 FGDs (Table 1). The smallest FGD had two participants and the largest had six. The study protocol was approved by institutional review boards at the Johns Hopkins School of Public Health in the US and at the Instituto Nacional de Salud Pública in Mexico.

**TABLE 1:** Focus group participant characteristics (N=56). Pack Appeal, Mexico, 2018.

Group	Age	Gender	SES	Smoking status
FGD-1 (n = 3)	Adolescent	Female	Low	Smoker
FGD-2 (n = 6)	Adolescent	Female	Low	Smoker
FGD-3 (n = 3)	Adolescent	Female	Mid/high	Smoker
FGD-4 (n = 2)	Adolescent	Female	Low	Non-smoker
FGD-5 (n = 4)	Adolescent	Female	Low	Non-smoker
FGD-6 (n = 5)	Adolescent	Female	Mid/high	Non-smoker
FGD-7 (n = 4)	Adolescent	Male	Low	Smoker
FGD-8 (n = 5)	Adolescent	Male	Mid/high	Smoker
FGD-9 (n = 3)	Adolescent	Male	Low	Non-smoker
FGD-10 (n = 4)	Adolescent	Male	Mid/high	Non-smoker
FGD-11 (n = 4)	Young adult	Female	Low	Smoker
FGD-12 (n = 4)	Young adult	Female	Mid/high	Smoker
FGD-13 (n = 2)	Young adult	Male	Low	Smoker
FGD-14 (n = 3)	Young adult	Male	Low	Smoker
FGD-15 (n = 4)	Young adult	Male	Mid/high	Smoker

### Participant Recruitment

Participants were enrolled by household-based recruitment throughout Mexico City. The city was divided into quadrants: North, South, Center, and West, and within each quadrant, a three-stage sampling procedure was applied. First, six neighborhoods were selected by probability sampling proportional to the number of occupied dwellings. Next, three blocks were selected in each region following the same procedure. Finally, a systematic household skipping protocol was employed: starting at a randomly selected point, recruiters visited every 5^th^ household. Additional information on recruitment is given in Supplementary Material Online Resource 1.

Household recruitment consisted of: 1) introducing the study, 2) eligibility screening, and 3) scheduling. Adolescents were screened only with the permission of their parent. Those who reported smoking at least one cigarette in the past 30 days were considered smokers and those who reported never having smoked cigarettes were considered non-smokers. Young adults were eligible if they reported smoking 100 cigarettes over their lifetime and at least one cigarette in the past seven days [17, 18]. Eligible individuals were scheduled to participate in the corresponding FGD at a later date. Adolescents’ parents provided informed consent before scheduling.

### Focus Group Procedures

Participants were individually engaged in the assent/informed consent process prior to the FGD start, which included permission to video record the session. FGDs were moderated using a semi-structured discussion guide. This was the first encounter between moderators and participants.

#### Pack Sorting Exercise

**FIGURE 1:**
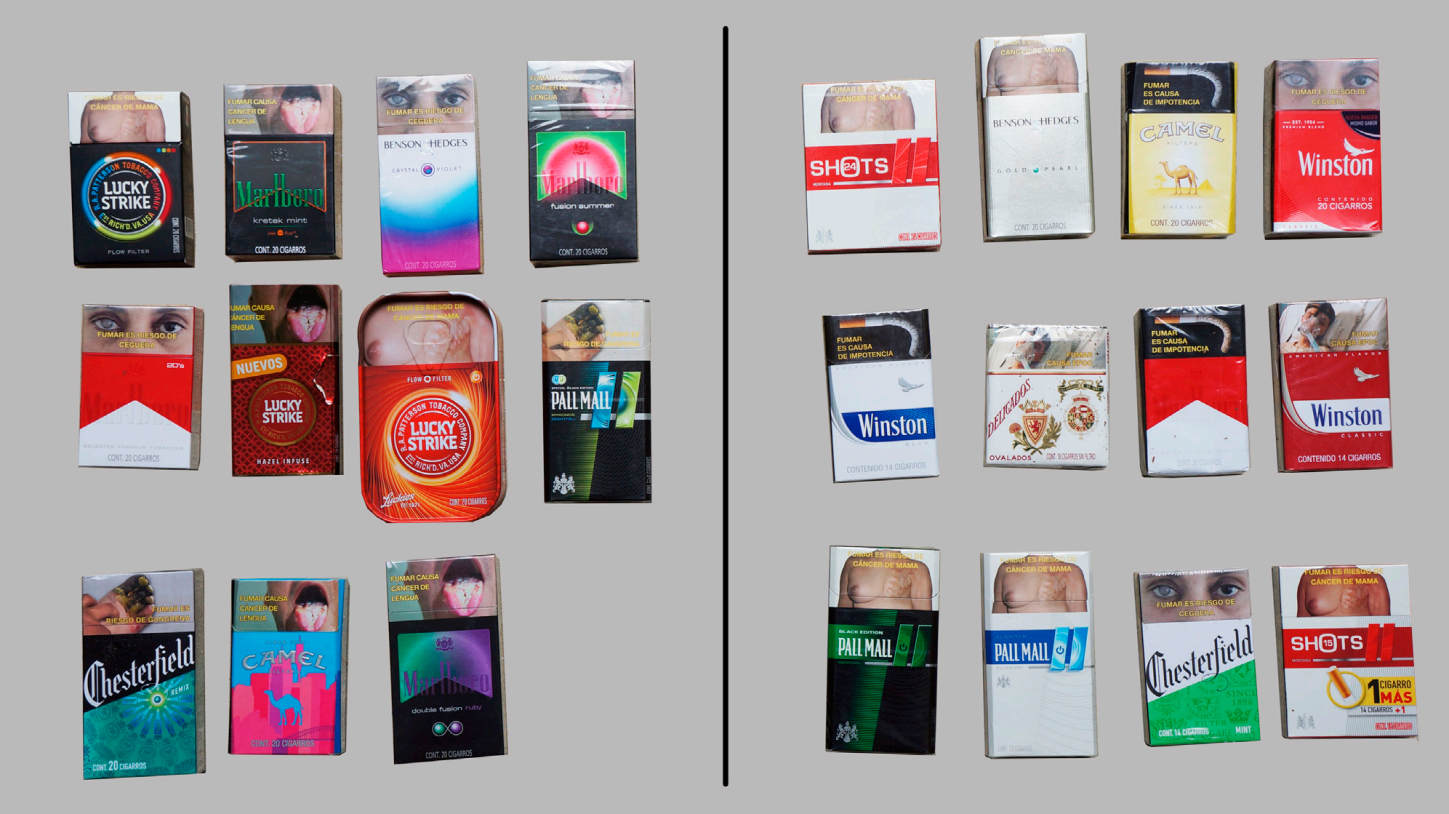
Packs in the initial appealing **(left)** and unappealing **(right)** groups. Pack Appeal, Mexico, 2018.

Participants were first given 23 cigarette packs and told they were divided into groups that were believed to be appealing and unappealing to young people (Figure 1). These initial groupings were determined by the research team using knowledge from the literature and findings from the pilot study. The packs used in the FGDs were bought in Mexico City shortly before the sessions to reflect products on the market at the time. Participants were asked to review the groupings and make changes in accordance with their shared perceptions of packaging appeal. Final regroupings were photographed.

#### Follow-Up Discussion

As the pack sorting was occurring and afterward, moderators guided participants in a discussion of the features, concepts, and content of the appealing and unappealing packs. Finally, each participant selected a favorite and least favorite pack, explaining the reasons behind these choices and describing the perceived audience(s) for each selection. Following completion of the FGDs, participants completed a brief questionnaire on demographics and smoking history, attitudes, and beliefs; and, moderators gave each participant a gift card of $400 MXN (∼$20 USD) and information on smoking cessation.

### Focus Group Coding and Analysis

Discussions were video recorded, professionally transcribed in Spanish, and translated into English. The first step of the analysis consisted of a review of the photographs from the final regroupings of each FGD to observe if and how participants changed the original groupings. Then, transcripts from the FGDs were coded using MAXQDA 2018 [19]. Video recordings supported the analysis by providing insight into the sorting process. The codebook consisted of both a priori codes based on features and appeals identified by previous research (including pack size, brand, color, and flavor) [20], as well as the domains covered in the discussion guide (perceived target audience, least/favorite pack, brand recognition, health warning labels, tobacco attitudes, and smoking habits). Two independent coders (LPL and GG) reviewed three transcripts to refine and expand the codebook to include other features mentioned by participants. Following initial coding, all transcripts were coded by one researcher (GG) and, once completed, data were subjected to thematic analysis. Identified themes were compared within and across groups and focused on the discussion of the most appealing features of the cigarette packs.

## Results

Regrouping decisions were similar across all FGDs. Two groups of adolescents (low-SES, female non-smokers and mid/high-SES, male non-smokers) and one group of male young adults (low-SES) did not regroup any packs. The maximum number of packs moved between groups was five. Five FGDs created a third grouping, increasing the number of movements.

Our analysis showed that three different pack features contributed to the maintenance of groupings or regrouping: color, flavor, and promotion. The discussion of color and flavor often overlapped; therefore, they are presented together. We do not often distinguish between flavored cigarettes and flavor capsule cigarettes in our results; however, most packs in our sample were flavor capsule cigarettes reflecting the Mexican market [21]. In each section, we present the results of the analysis of the pack movements, followed by the discussion of why packs were moved or not. To conclude, we report the results of the discussion on perceived packaging audience.

### Color and Flavor

#### Analysis of the Groupings

**FIGURE 2:**
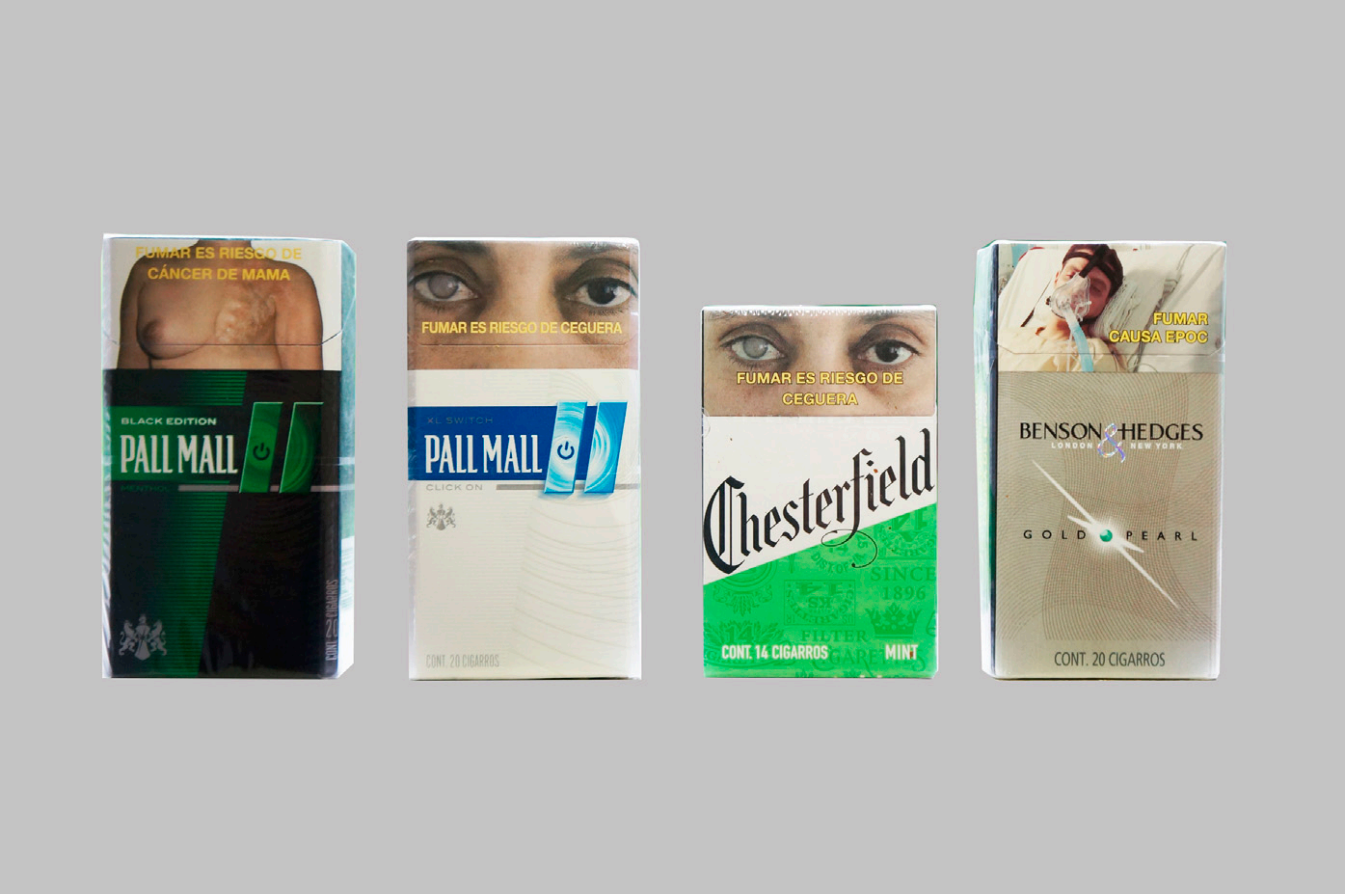
Packs with bold, contrasting colors and/or flavors moved from the unappealing pile to the appealing. Pack Appeal, Mexico, 2018.

Color and flavor emerged from the discussions and actions taken as important features that serve to increase or decrease pack appeal. Across the 12 FGDs that regrouped from the original pack groupings, four packs featuring flavor and/or bold, contrasting colors were moved from the unappealing to the appealing group (Figure 2). For example, the pack *Pall Mall Black Edition* was moved from the unappealing to the appealing group by seven groups of adolescents (low, mid/high-SES female smokers, two low-SES female non-smokers, low, mid/high-SES male smokers, low-SES male non-smokers) and three groups of young adults (mid/high-SES females, low, mid/high-SES males).

During the discussion, adolescents and young adults further elaborated that certain color combinations, shades, and brightness increase appeal and draw attention whereas others have the opposite effect.

The black is (…) a background for more eye-catching, brighter colors (male adolescent smoker, mid/high-SES).

The color combination [pink and blue Camel] is what stands out (…) because you focus on it (male young adult, mid/high-SES).

[T]hey’re very pale colors [“traditional” yellow Camel] and they don’t draw attention (female adolescent smoker, low-SES).

Participants discussed why certain colors were more appealing than others, with some indicating that they were attracted to their favorite color on the pack.

At first, more than anything, it was the purple color that got my attention, it’s my favorite. I see something purple and I always like to try it (…). I try to experiment all the eye-catching things I see, which is the intention I suppose (male young adult, low-SES).

#### Intersection of Color and Flavor

**FIGURE 3:**
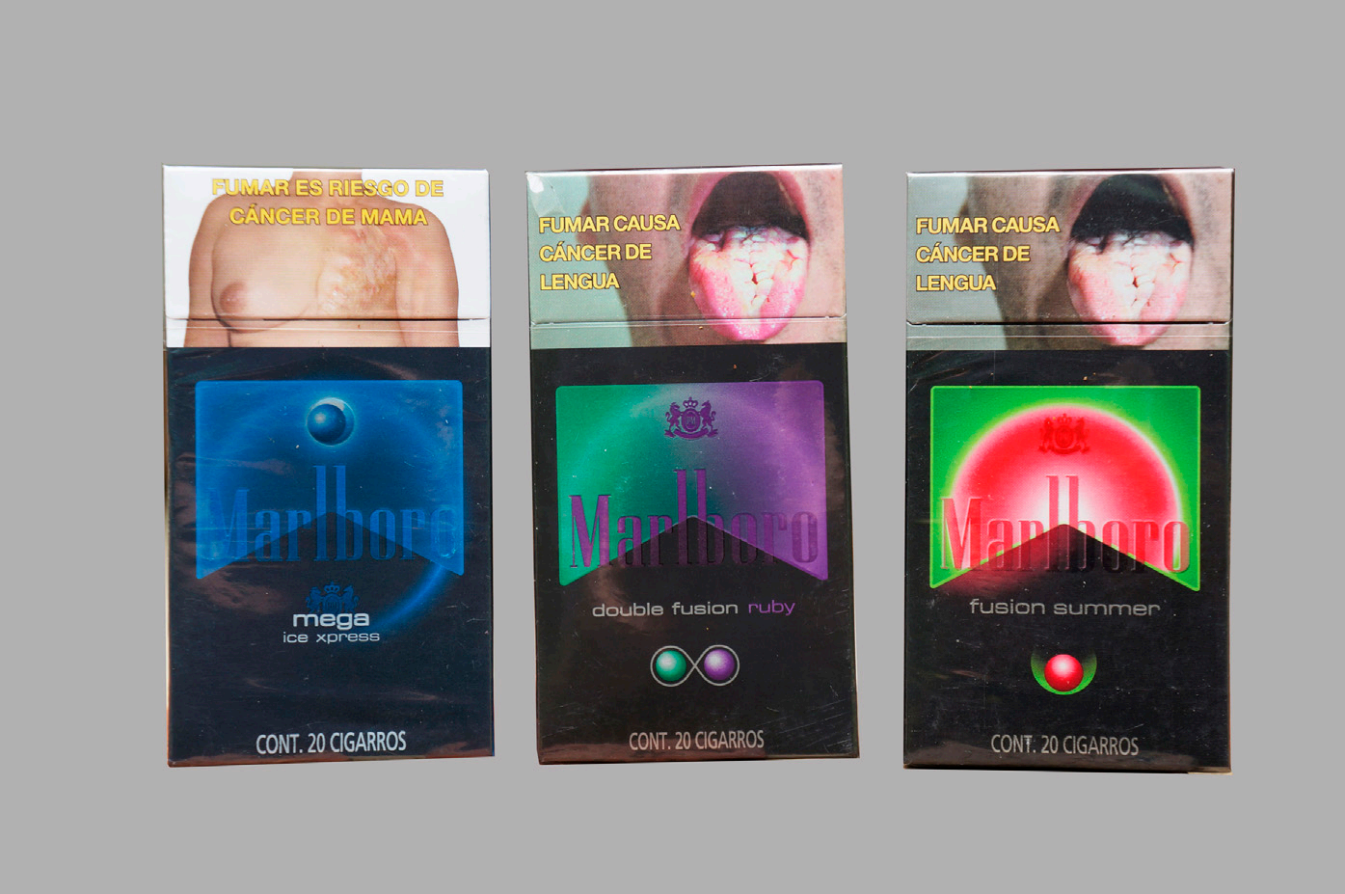
Packs of Marlboro with different flavors and capsule formats; the Marlboro Fusion Summer was commonly referred by participants as the “Marlboro watermelon” due to its color and design. Pack Appeal, Mexico, 2018

Colors on the pack were discussed as conveying addition of flavor (including via flavor capsules) to cigarettes (Figure 3) especially in the groups of smokers and mid/high-SES male adolescent non-smokers.

You can imagine what the flavor is because of the colors (female adolescent smoker, low-SES).

Because the colors pop and you can say, “Oh, it’s watermelon!” [Marlboro Fusion Summer] (female adolescent non-smoker, mid/high-SES).

The availability of different cigarette flavors increased the attractiveness of the pack, which was conveyed by multiple colors on the pack.

When the pack says “flavored” and it has some specific color, I relate it with flavor. For example, blue with mint, green with spearmint (female adolescent smoker, mid/high-SES).

#### Experiencing Flavors and Flavor Capsules

Moreover, flavors were associated with the experience of trying different things.

It’s like fascination, what they’ll taste like, right? It’s something new, you know? (female young adult, low-SES).

Flavors were especially appealing to smokers because they modified the taste and smell of cigarettes. This sensory experience might result in an increased pleasant sensation and interest in trying other flavors.

I started to like these [Pall Mall Mykonos Nightfall] because my grandmother bought them and I smelled the scent of the cucumber flavor capsule and said, “I want to try them,” (male adolescent smoker, mid/high-SES).

First, in part because of the blending design, the colors, the capsule that says it is just one [capsule that] has two flavors, and the experience that they are fresh [Benson and Hedges Crystal Violet] (female adolescent smoker, mid/high-SES).

In general, participants easily identified the existence of flavor capsules on the pack and knew that they worked by releasing flavor when crushed, inciting participants’ curiosity and desire to try the many flavors.

I don't know why the capsule appeals to me, I feel I want to know what it tastes like (female adolescent smoker, low-SES).

Discussions on the appeal of the capsules were slightly more predominant among male and female smokers of all ages compared with non-smokers. In addition, mid/high-SES female smokers specifically discussed that the presence of double-capsules further increased the appeal of the pack because it changed their smoking experience.

You crush one flavor at the beginning, and then halfway through you crush the other one to taste. At least that’s what I do (female young adult, mid/high-SES).

#### Flavors and Sensory Perceptions

Discussions also revealed that flavored cigarettes are often perceived to be “smoother.” Moreover, participants across all groups indicated that a milder taste is something appealing to youth. Cigarette strength was connected to packaging design, particularly colors and flavors.

It does tell me it has a strong taste, because of the presentation [Delicados] (male young adult, low-SES).

A lot of young people (…) tell me, “I buy these because they have a capsule and they taste better.” They don’t taste as strong as Marlboro or Shots. And besides, I think that’s why these attract young people more, because of the flavors and the colors (male adolescent smoker, mid/high-SES).

Female young adults (mid/high-SES) shared a unique perception that flavored cigarettes appeal to youth because flavors would mask the smell of tobacco.

Young people (…) smoke it because it's smooth. Sometimes they go out to eat or something like that, they smoke it and go back to the office without any smell (female young adult, mid/high-SES).

### Promotional Packs

#### Analysis of the Groupings

**FIGURE 4:**
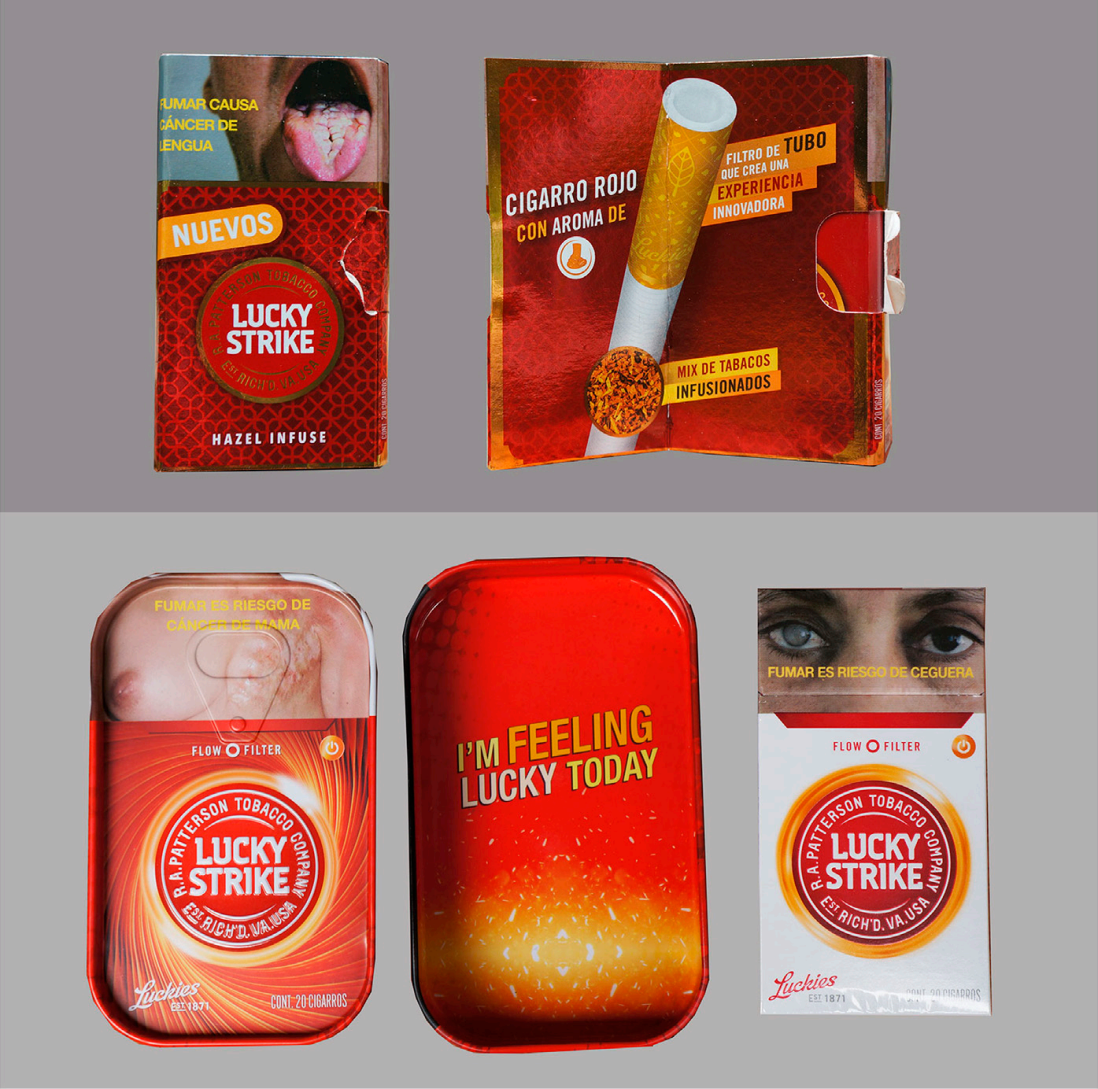
Examples of promotional packs in the sample. Pack Appeal, Mexico, 2018.

The group of low-SES female adolescent non-smokers created a third group classifying promotional packs, such as the *Shots 14 Cigarettes +1*, the *Lucky Strike* metallic box, and the *Lucky Strike* with a shiny metallic booklet sleeve and the word “New” (*Nuevos*) on it (Figure 4). Low-SES male adolescent non-smokers also moved the *Shots 14 Cigarettes +1* to the appealing pile. The *Lucky Strike* metallic box was kept in the appealing pile by all groups, but one of the groups of low-SES female adolescent smokers.

#### Packaging Communicating Cigarette Promotion

In general, participants indicated that promotional packs sparked their curiosity. In the case of the *Lucky Strike* metallic box, most groups found it appealing because they could use the box for other things, such as a cigarette case (young adult females, low-SES), collection (young adult males, mid/high-SES), and to storing money and jewelry.

Because lots of guys want the box, it appeals to them (male adolescent smoker, low-SES).

My dad used to buy a lot of this kind of box (…) [although] his normal pack [was] (…) the usual Marlboro, (…) he collected them (male young adult, mid/high-SES).

Other features communicating promotion on the pack, such as one free cigarette, were particularly appealing to mid/high-SES adolescent female smokers and low-SES adolescent female and male non-smokers.

It’s more appealing to buy one of these, because it gives you an extra cigarette (male adolescent non-smoker, low-SES).

### Perceived Packaging Audience

Participants described the perceived audience for the packs, reinforcing notions of self-identification (or lack of it) with certain cigarette packs. A common theme among all groups was that colored and flavored packs are more appealing and used more frequently by young women.

I feel that this one would go into the appealing, since the colors appeal more to a woman who smokes because of the colors, and besides (…) what I have heard and seen is that, really, the capsule cigarettes are for women (male adolescent non-smoker, mid/high-SES).

[M]y guy friends say they don’t like the capsules; they prefer them without anything because they say they’re for girls (female adolescent smoker, low-SES).

The “unappealing packs” were associated especially with established, middle-age and older male smokers, for whom flavors would not be appealing.

I think one [pack] that has more colors and things like that is younger. Well, I suppose that if I were an adult, I no longer would focus so much on the appearance of a pack, on the colors (…), because I have smoked many, I would just go for the one that I want, without caring so much about the appearance of the pack (female adolescent smoker, mid/high-SES).

You arrive at a family reunion and tell your fifty-year-old uncle, “Give me a cigarette.” [Y]ou’re going to get those [unappealing packs] (female young adult, low-SES).

#### Perceptions of Packaging and Buying Choices

**FIGURE 5:**
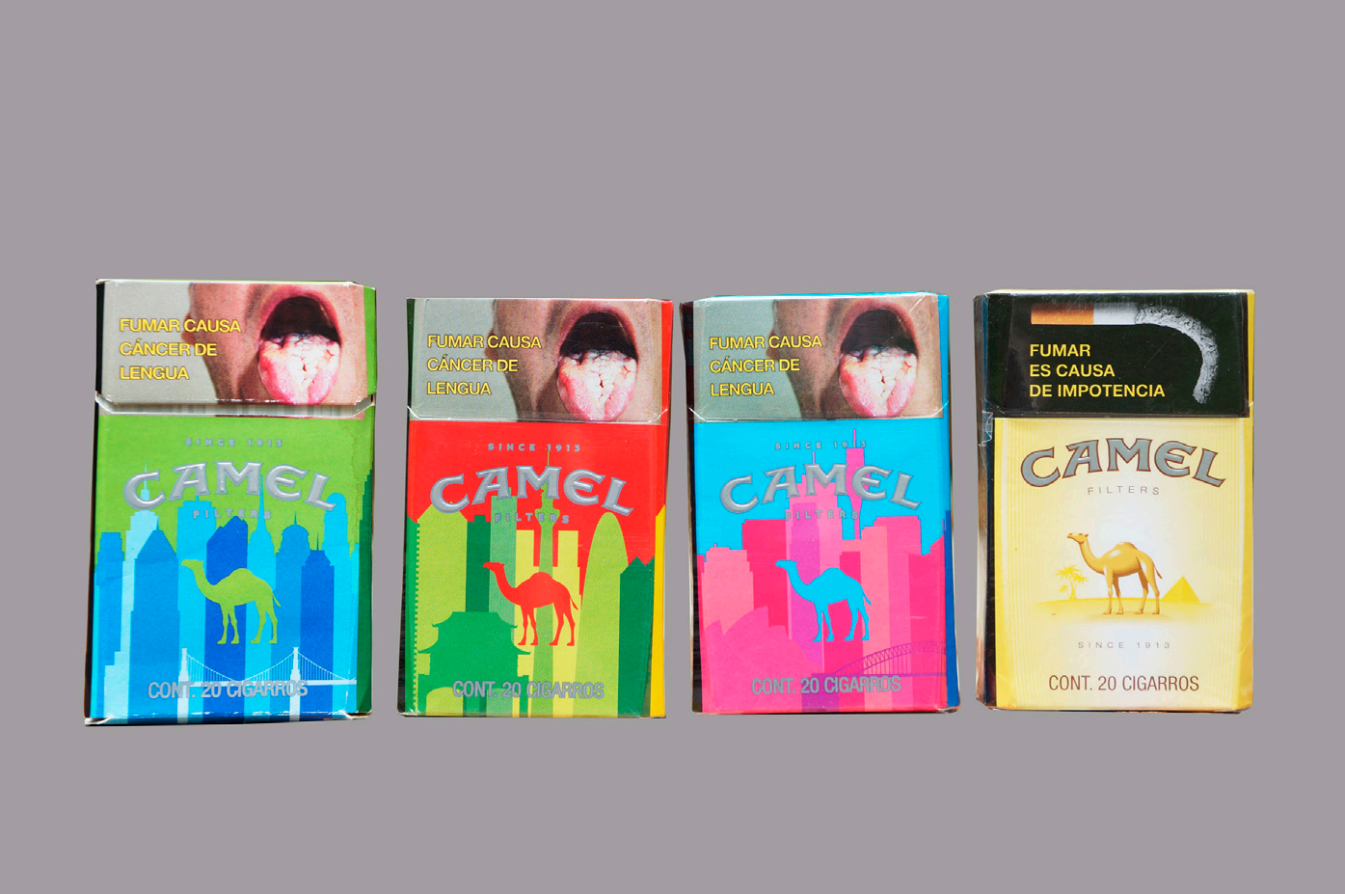
Colorful Camel packs in contrast with the traditional yellow Pack Appeal, Mexico, 2018.

Overall, participants recognized the importance of the design and that changing the design of a known pack might increase its appeal (Figure 5).

I like these. I would try them. I don’t know if they're the same as this one [“traditional” yellow Camel], (…), but I would buy it because I like the pack (female young adult, low-SES).

Despite recognizing that packs are designed to increase appeal, low-SES male adolescent smokers and young adults minimized the importance of the design when purchasing their own cigarettes.

In the end, I think they aren’t classified by design, or the pack or anything, it’s just (…) a cigarette, and you like the cigarette, you like menthol—you go for a menthol (male young adult, low-SES).

The packs are not what gets our attention but rather the flavor of the cigarette or what the cigarettes are made of (female young adult, low-SES).

Yet, young adults were aware that packs are designed to potentially target them.

They [cigarette packs] are getting trendier, and they are thinking more right now about young people, because we as young people are really into this. It’s all about the colors. So, they say, well this one is catching on, so we’ll put out another one like it (female young adult, mid/high-SES).

## Discussion

Through a series of FGDs with adolescents and young adults, our study examined how specific pack features may increase or decrease cigarette pack appeal. Overall, our results showed that bold, contrasting colors, flavors (including flavor capsules), and promotion increased the appeal of packs and the desire to try. Packs with those features were perceived to be used and designed for youth.

All FGDs included discussion about how bright, shiny colors increased the appeal and caught consumers’ attention, as reported by other studies [6, 9]. However, in contrast with previous studies where dark colored packs (such as black and brown) were associated with stronger tobacco flavor [22] and were deemed less appealing [6, 9, 23], participants grouped the black packs in our sample in the appealing pile also because they were perceived as smoother. This could be explained by the fact that the color black provided a contrasting background to bright and vibrant colors as described by some of the participants, which conveyed the addition of flavor to cigarettes.

Participant smokers in particular discussed the association between color and flavor and how flavor altered their smoking experience because of the taste and smell of cigarettes. The availability of different flavors and multiple flavors in the same pack increased the desire to try the product. At the same time, young adults diminished the importance of packaging design in comparison to their smoking experience and the taste and smell of cigarettes. This apparent contradiction highlights the success of tobacco companies in using color to influence consumers’ perception of the product’s physical characteristics [22]. Regardless of smoking experience, all participants perceived flavored cigarettes to be smoother, increasing the appeal to youth [10].

The addition of flavor to cigarettes was a key design feature that increased appeal across all FGDs. Participants easily identified the flavor capsules on the pack and knew how they worked, as previously reported [21]. While in places like the US flavor capsules have been associated with the premium cigarette market, a variety of flavor capsules are available in the discount market segment in Mexico [24], which may explain why groups identified them as appealing regardless of SES. It is worthwhile mentioning that flavor capsules are available in several discount markets across Latin America, where the capsule market has been reportedly growing [25].

In our study, female young adults particularly stressed the contribution of capsules to product appeal. These findings are consistent with a study in Chile that identified young people under the age of 25 years and females as the main consumers of flavor capsules, regardless of SES [26], and one in Mexico that found age and female gender as predictors for liking capsule cigarettes [24]. Young women also deliberated on capsules being particularly appealing because they provided different options and combinations when smoking besides being smoother and reducing the odor of cigarettes. Similar findings have been reported among female Scottish youth [27].

Despite the Mexican law prohibiting promotional items with the name or logo of tobacco companies as well as incentives to purchase tobacco [12], some packs in our sample presented unique and innovative designs communicating promotions (one free cigarette), limited edition items (branded metallic box) or novelty packaging features (shiny sleeve with word new). These elements increased participants’ curiosity about the product especially among adolescent smokers and non-smokers. These findings are congruent with other studies that have reported the effects of design innovation in increasing appeal and susceptibility to smoke [22, 28, 29]; similarly, reviews of tobacco industry documents showed their awareness of how pack innovation could influence consumer behavior [30]. Moreover, they reinforce the need of considering the cigarette pack as a marketing tool and a form of advertisement that must be regulated as part of a comprehensive TAPS ban. This can be a way of minimizing the effects of packaging design in countries that have not adopted plain packaging.

### Strengths and Limitations

Our findings should be regarded within the limitations of the study. First, given the inclusion of adolescents who did not know each other, some groups were not very talkative, despite moderators’ experience and use of multiple prompts. Second, the pack sorting exercise began with groupings sorted *a priori* by the research team. We opted for this approach to provide structure to the exercise: a completely organic sorting process, with participants collectively producing their own “appealing” and “unappealing” groups would have been time consuming and burdensome for participants in this particular study. However, the *a priori* groups might have influenced participants’ classification and perceptions of appealing and unappealing features. Future studies with the capacity for an organic pack sorting process would add additional insight to our findings. Finally, only residents from Mexico City were recruited; therefore, our findings might not be generalizable to other geographical settings in Mexico. Nevertheless, this study has several strengths. The focus group design allowed insight into the factors that youth use when assessing whether packs are appealing. The availability of video allowed us to observe participants’ interactions with specific packs and identify packs being discussed. Our instruments were refined following a pilot study conducted in Baltimore, United States. Based on those findings (not reported here), we organized the FGDs by smoking status in addition to gender and SES to maximize the possibility that participants would feel comfortable sharing their opinions. The small number of participants in each group permitted the contributions of all individuals.

### Implications for Policy and Practice

This study found that certain design features of cigarettes packs and especially color of the package, flavor and promotion are associated with increased product appeal to adolescents and young adults in Mexico City. This reinforces the need for adopting plain packaging as a key step to reduce pack appeal [2, 6, 7, 10, 28, 31] and prevent initiation [8, 27, 31], especially among youth. If this were combined with stronger TAPS restrictions, such as banning the display of cigarette packs at the POS, which is still allowed in Mexico, tobacco control in the country would be strengthened. Even with plain packaging, however, to the extent that flavored cigarettes are available, so this might facilitate young smokers in practices of initiation and social smoking, as well as reducing likelihood of cessation attempts. A comprehensive flavor ban is another key step to prevent youth smoking.

## Data Availability

The datasets presented in this article are not readily available because Data are available upon reasonable request. Requests to access the datasets should be directed to GG, gribeir2@jhu.edu.
