# Supplementary material for: “It’s all About the Colors:” How do Mexico City Youth Perceive Cigarette Pack Design
Source: Int J Public Health. 2021 Mar 10;66:585434. doi: 10.3389/ijph.2021.585434 (PMC8565279; doi:10.3389/ijph.2021.585434)
Supplement: Supplementary file 1 [file DataSheet1.PDF]

Journal International Journal of Public Health

Article name “It's all about the colors”: how do Mexico City youth perceive cigarette pack design

Online Resource 1 - Household-based recruitment

For this study, the universe is comprised of the occupied dwellings in the Mexico City area. The population groups are adolescents (ages 13 to 17 years) and young adults (ages 18 to 24 years).

To select the sample, Mexico City was divided into four quadrants: North, South, Center and West, with each one considered as a stratum. A three-stage sampling procedure was then applied in each quadrant.

First, a total of **6** Basic Geostatistical Areas were selected (AGEB). The AGEB is a division within each delegation in Mexico City and serves for census and survey field work (i.e. neighborhood), which constitutes a good sampling frame, since they cover the whole country. Neighborhoods were selected by probability sampling proportional to the number of occupied dwellings (i.e., neighborhoods with more occupied dwellings had a higher probability of selection). The selection probabilities in this stage is given by:

$$M_{ir} = \frac{6\ C_{ir}}{C_r}$$

Where:

M_{ir}: Is the selection probability of i-th neighborhood in region r of Mexico City.

C_{ir}: Is the total number of dwellings in the i-th neighborhood in region r

C_r: Is the total number of dwellings in region r in Mexico City.

Second, a total of three blocks in each region was selected also by probability proportional to the number of occupied dwellings. The selection probability of a block is given by the following equation:

$$M_{ijr} = \frac{3\ C_{ijr}}{C_{ir}}$$

Where:

M_{ijr}: Is the selection probability of j-th block from the i-th neighborhood in region r of Mexico City.

C_{ir}: Is the total number of dwellings in the i-th neighborhood in region r of Mexico City

C_{ijr}: Is the total number of dwellings in the j-th block in the i-th neighborhood in region r of Mexico City

During field work, each block was visited in order to count the number of occupied dwellings. Then a random number between 1 and the total number of occupied dwellings was obtained from a random number table. This number was used to select the first dwelling of a segment of five dwellings, which was then used to continue following a systematic household skipping protocol (i.e., after randomly selecting the first dwelling, recruiters visited every 5th dwelling). The selection probability in this stage is given by the following equation:

$$M_{ijkr} = \frac{5}{C_{ijr}}$$

Where:

M_{ijkr}: Is the selection probability of k-th dwelling in the j-th block in the i-th neighborhood in region r of Mexico City

C_{ijr}: Is the total number of Dwellings in j-th block in the i-th neighborhood in region r of Mexico City

In this way the final probability of a dwelling is given by:

$$P_{ijkr} = \frac{6\, C_{ir}}{C_r} * \frac{3C_{ijr}}{C_{ir}} * \frac{5}{C_{ijr}} = \frac{90}{C_r}$$

The same sampling scheme was used for each of the four regions in which Mexico City was divided.
